# A data citation roadmap for scholarly data repositories

**DOI:** 10.1038/s41597-019-0031-8

**Published:** 2019-04-10

**Authors:** Martin Fenner, Mercè Crosas, Jeffrey S. Grethe, David Kennedy, Henning Hermjakob, Phillippe Rocca-Serra, Gustavo Durand, Robin Berjon, Sebastian Karcher, Maryann Martone, Tim Clark

**Affiliations:** 1 DataCite, Hannover, Germany; 2 Institute for Quantitative Social Science, Harvard University, Cambridge, MA USA; 3 University of California San Diego, La Jolla, CA USA; 4 University of Massachusetts Medical School, Worcester, MA USA; 5 European Bioinformatics Institute (EMBL-EBI), European Molecular Biology Laboratory, Hinxton, Cambridgeshire UK; 6 Oxford e-Research Centre, University of Oxford, Oxford, UK; 7 Standard Analytics, New York, NY USA; 8 Qualitative Data Repository, Syracuse University, Syracuse, NY USA; 9 University of Virginia School of Medicine, Charlottesville, VA 22903 USA

**Keywords:** Databases, Computational platforms and environments, Databases, Data publication and archiving

## Abstract

This article presents a practical roadmap for scholarly data repositories to implement data citation in accordance with the Joint Declaration of Data Citation Principles, a synopsis and harmonization of the recommendations of major science policy bodies. The roadmap was developed by the Repositories Expert Group, as part of the Data Citation Implementation Pilot (DCIP) project, an initiative of FORCE11.org and the NIH-funded BioCADDIE (https://biocaddie.org) project. The roadmap makes 11 specific recommendations, grouped into three phases of implementation: a) required steps needed to support the Joint Declaration of Data Citation Principles, b) recommended steps that facilitate article/data publication workflows, and c) optional steps that further improve data citation support provided by data repositories. We describe the early adoption of these recommendations 18 months after they have first been published, looking specifically at implementations of machine-readable metadata on dataset landing pages.

## Introduction

The Joint Declaration of Data Citation Principles (JDDCP) published in 2014^1^ and endorsed by a large number of scholarly and academic publishing organizations, lays out a set of principles on purpose, function and attributes of data citations. The first of these principles stresses that data should be considered legitimate, citable products of research^2^. The JDDCP condenses the results of substantial prior studies on science policy and practice^3–5^.

The JDDCP intentionally focuses on data citation principles, as the implementation of these principles will differ across disciplines and communities. The roadmap presented here aims to provide practical guidance for repositories on implementing these data citation principles with a focus on life sciences, based on earlier work in this area, in particular Starr *et al*.^6^ and Altman and Crosas^7^, and are consistent with recent recommendations regarding data, code and workflows^8,9^. These recommendations for data repositories complement the DCIP project recommendations for publishers^10^ and for globally unique resolution of Compact Identifiers^11^. While related recommendations might differ in implementation detail, we do not know of any conflicting recommendations that the reader should be aware of.

Data repositories play a central role in data citation, as they provide stewardship and discovery services to find data, give persistent access to the data being cited, and provide unique identifiers and metadata needed for data citation. For data citation, repositories need to work closely with a variety of stakeholders, including publishers, reference manager providers, data users, and of course researchers. Data citation practices and technologies supported by repositories will substantially assist development of new data discovery indexes such as DataMed^12^ and Google Dataset Search (https://toolbox.google.com/datasetsearch).

## Results

The guidelines are grouped into three phases: required, recommended and optional. Implementing these guidelines takes time and resources, it is therefore not only critical to provide specific guidelines, but also to give guidance on priorities: work needed to support the Joint Declaration of Data Citation Principles (required phase), additional work to facilitate article/data publishing workflows in collaboration with publishers (recommended phase), and extra work to support data citation that can be done by data repositories (optional phase). The Guidelines are summarized in Table 1, and are discussed in detail in the text following the table.

**Table 1:** Guidelines for Repositories.

Level	#	Guideline
Required	1	All datasets intended for citation *must* have a globally unique persistent identifier that can be expressed as an unambiguous URL.
	2	Persistent identifiers for datasets *must* support multiple levels of granularity, where appropriate.
	3	The persistent identifier expressed as an URL *must* resolve to a landing page specific for that dataset, and that landing page must contain metadata describing the dataset.
	4	The persistent identifier *must* be embedded in the landing page in machine-readable format.
	5	The repository must provide documentation and support for data citation.
Recommended	6	The landing page *should* include metadata required for citation, and ideally also metadata facilitating discovery, in human-readable and machine-readable format.
	7	The machine-readable metadata *should* use schema.org markup in JSON-LD format.
	8	Metadata *should* be made available via HTML meta tags to facilitate use by reference managers.
	9	Metadata *should* be made available for download in BibTeX and/or another standard bibliographic format.
Optional	10	Content negotiation for schema.org/JSON-LD and other content types *may* be supported so that the persistent identifier expressed as URL resolves directly to machine-readable metadata.
	11	HTTP link headers *may* be supported to advertise content negotiation options

Details of each recommendation follow, with examples.

### Persistent identifiers

A data citation *must* include a persistent method for identification that is machine actionable, globally unique, and widely used by a community (**JDDCP**, principle #4). The use of the persistent identifier should follow community best practices^6,13–16^. For implementation by data repositories, this means:

- **Persistent method for identification**. Unique identifiers, and metadata describing the data, and its disposition, *must* persist–even beyond the lifespan of the data they describe (**JDDCP**, principle #6). As an extension to this principle, data repositories should make provisions to keep unique identifiers and metadata available beyond the lifespan of the data or repository, ideally in a well-recognized and accepted standard metadata format.
- **Machine actionable**. The persistent identifier *must* be understood, and be resolvable, as an HTTP URI in accordance with IETF RFC 3986^16,17^, including support for content negotiation^18^.
- **Globally unique**. The identifier *must* use a prefix (namespace) if the identifier character string is only unique within a particular database, e.g. an accession number; and the prefix must be registered with a robust, institutionally stable global resolver such as the identifiers.org system at EMBL/EBI^11^.
- **Widely used by a community**. The persistent identifier must be widely used in the community. For the life sciences this includes accession numbers, in combination with the database name for global uniqueness.

### Persistent identifier granularity

Persistent identifiers for datasets must support multiple levels of granularity to support both the citation of a specific version and/or individual dataset, as well the citation of an unspecified version of a dataset and/or a collection of primary data. The levels of granularity supported by persistent identifiers must be documented.

In many domains, primary data is uniquely identified and cited as a collection of potentially many individual items. At the same time, these individual items need their own unique identifiers to support later reuse and recombination into different sets while maintaining the ability to cite the constituent data elements. An example is in the field of neuroimaging, where individual subject scans using a given imaging modality are the lowest level at which objects will be identified, while the primary publication will cite a collection level unique identifier. This imposes a requirement that lower-level identifiers need to be able to be grouped via a collection identifier and accessed as set elements from the overall collection landing page 18. Another example is the BioStudies database^19^, which can provide storage for all the underlying data links and files for a publication.

Only in circumstances where multiple levels do not inherently exist in the data, i.e. no collections or other groupings exist, may this requirement be waived.

### Landing pages

**Fig. 1:**
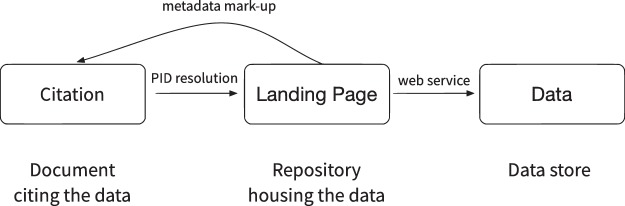
Generic data citation - relationships of the citation reference, repository landing page and underlying data.

The persistent identifier expressed as HTTP URL must resolve to a specific landing page for that dataset or dataset collection. The persistent identifier expressed as HTTP URL must not resolve to the data itself 6, or to other representations of the metadata, unless special protocols such as content negotiation are used (see guideline 7 below). Relationships of the citation reference, repository landing page and underlying data are shown in Fig. 1.

The landing pages *must* provide metadata with additional information about the dataset, and include links for accessing the dataset itself. The landing page *should* provide definitive information, including metadata, on how the dataset should be cited, other descriptive information about the dataset, as well as data accessibility and licensing information. Repositories should provide a landing page for every dataset or collection of datasets intended to be cited, which could be single entries, sets of entries, the entire repository or a curated database^6^.

Reference to a statement describing the data and metadata persistence policies of the repository should also be provided at the landing page. Data persistence policies will vary by repository but should be clearly described, for example (using text template from^6^):

*“[Organization/Institution Name] is committed to maintaining persistent identifiers in [Repository Name] so that they will continue to resolve to a landing page providing metadata describing the data, including elements of stewardship, provenance, and availability*.

*[Organization/Institution Name] has made the following plan for organizational persistence and succession: [plan].”*

**Fig. 2:**
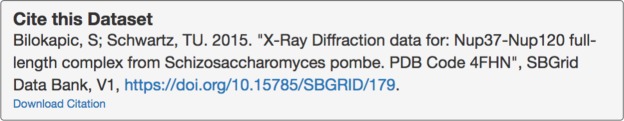
Providing information about how a dataset should be cited, with download link for citation (in BibTex or other standard bibliographic reference manager format).

Figure 2 provides an example for how “Cite this Dataset” information can look in a landing page.

### Persistent identifiers on landing pages

To verify that a persistent identifier resolves to a correct landing page, the persistent identifier *must* be embedded in the landing page in human-readable and machine-readable formats. This enables checks that the persistent identifier properly resolves to a landing page describing that identifier, and enables basic data citation by reference managers, and minimal validation by the publisher of persistent identifiers cited in documents. The persistent identifier should be found somewhere on the landing page, but is ideally embedded in schema.org markup and/or using HTML meta tags.

Example schema.org/JSON-LD

<application type="application/ld+json">

{"@id": "https://doi.org/10.5061/dryad.q447c/3"}

</application>

Example HTML meta tags

<meta name="DC.identifier" content="https://doi.org/10.5061/dryad.q447c/3">

### Documentation and author support

The repository *must* provide documentation about how data should be cited, how metadata can be obtained, and who to contact for more information. This documentation should follow the recommendations in this document, the DCIP Data Citation Primer^20^, community recommendations provided by a number of organizations, but should also address the specifics of that particular data repository.

### Metadata on landing pages

Landing pages should provide metadata required for data citation in both human- and machine-readable format, and should be accessible without requiring authentication. The landing page should show the citation metadata in human-readable form, e.g. formatted in one or more citation styles common to the community in a Cite this Dataset field and, possibly, provide means of copying/downloading the citation as text. The landing page should also show all versions, or link to a page with version information. A visible link to machine-readable metadata should be provided.

The metadata elements needed for data citation are given in Table 2.

**Table 2:** Citation metadata for Data Repositories. Key:

Citation Metadata	Dublin Core^a^	Schema.org^b^	DataCite^c^	DATS^d^
Dataset Identifier	identifier	@id*	identifier	identifier
Title	title	name	title	title
Creator^**^	creator	author	creator	creator
Data repository or archive	publisher	publisher	publisher	publisher
Publication Date	date	datePublished	publicationYear	date
Version	*not available*	version	version	version
Type	type	type	resourceTypeGeneral	type

^a^Dublin Core Metadata Element Set (https://dublincore.org/documents/dces/);

^b^Dataset - Schema.org (https://schema.org/Dataset);

^c^DataCite Metadata Working Group^21^;

^d^Gonzalez-Beltran & Rocca-Serra^22,23^;

^*^name of ID field depends on schema.org serialization format, it is **@id** for JSON-LD;

^**^not all datasets will have “the main researchers involved in producing the data” (DataCite Schema), in which case the more generic “An entity primarily responsible for making the resource” from Dublin Core should be used, and this can also be an organization.

All metadata fields required for citation are part of Dublin Core (with the exception of *version*), the core schema.org specification, and by extension Bioschemas (https://bioschemas.org), as well as the DataCite and DATS metadata schemas^21–23^.

In addition to the metadata required for citation, it is recommended to provide additional metadata on landing pages – again in human-readable and machine-readable formats – that help with data discovery, as shown in Table 3.

**Table 3:** Important discovery metadata for Data Repositories. Key:

Discovery Metadata	Dublin Core	Schema.org	DataCite	DATS
Description	description	description	description	dataType dimension Material…*
Keywords	subject	keywords	subject	keywords
License	license	license	rights	license
Related Dataset**	isPartOf isVersionOf references	isPartOf citation	relatedIdentifier	isPartOf
Related Publication***	bibliographicCitation	citation	relatedIdentifier	publication

^*^DATS provides much more detailed metadata to describe a biomedical dataset;

^**^related datasets can have part/whole relations (IsPartOf, etc.), version relations (IsVersionOf, etc.) or reference relations (references);

^***^related publications reference a dataset published previously, reference a dataset published in parallel with the publication, or otherwise document a dataset.

The metadata standards Dublin Core, schema.org and DataCite by their very nature of being generic only provide some metadata helpful for discovery, while DATS can provide much more detailed information about a biomedical dataset. Further information can be found in the DATS specification^24^.

Information about related datasets should be provided where possible, as should information about related publications. They provide important information that can help with discovery. When a data repository knows about a publication citing a dataset, this information should be included in the metadata, complementing the information about the dataset found in the citing publication and enabling navigation between publication and dataset in both directions.

### Metadata on landing pages using schema.org/JSON-LD

All dataset landing pages *should* provide machine-readable metadata using schema.org markup in JSON-LD format. JSON-LD is the easiest way to represent schema.org metadata, and is also used to represent DATS metadata in schema.org format^23,24^. The JSON-LD should be embedded in the HTML page using a <script type="application/ld+json"> tag.

Examples

<script type="application/ld+json">

{"@context": "http://schema.org",

"@type": "Dataset",

"@id": "https://doi.org/10.3886/ICPSR08001.v2",

"name": "Cancer Surveillance and Epid

emiology in the

United States and Puerto Rico, 1973–1977 (ICPSR 8001)",

"author": "National Cancer Institute",

"publisher": "ICPSR - Interuniversity Consortium for Political and Social

Research",

"datePublished": "1984-05-03",

"dateModified": "2015-08-06T11:20:58Z",

"version": "v2",

"Description": "This dataset was produced as part of the Surveillance, Epidemiology, and End Results (SEER) Program to monitor the incidence of cancer and cancer survival rates in the United States, thus carrying out the mandates of the National Cancer Act. The SEER Program had several objectives: to estimate the annual cancer incidence in the United States, to examine trends in cancer patient survival, to identify cancer etiologic factors, and to monitor trends in the incidence of cancer in selected geographic areas with respect to demographic and social characteristics…"}

</script>

<script type="application/ld+json">

{"@context": "http://schema.org",

"@type": "Dataset",

"@id": "https://doi.org/10.2210/pdb5m95/pdb",

"name": "STAPHYLOCOCCUS CAPITIS DIVALENT METAL ION TRANSPORTER (DMT) IN COMPLEX WITH MANGANESE",

"author": [

{"@type": "Person",

"givenName": " I.A.",

"familyName": "Ehrnstorfer"},

{"@type": "Person",

"givenName": " E.R.",

"familyName": " Geertsma"},

{"@type": "Person",

"givenName": "E.",

"familyName": " Pardon"},

{"@type": "Person",

"givenName": " J.",

"familyName": " Steyaert"},

{"@type": "Person",

"givenName": " R.",

"familyName": " Dutzler"}],

"datePublished": "2016-11-30",

"publisher": "Protein Data Bank, Rutgers University",

"citation": [

{

"@type": "ScholarlyArticle",

"@id": "https://doi.org/10.1038/nsmb.2904"

}]}</script>

For further examples please use DataCite Search (https://search.datacite.org/), which has embedded schema.org/JSON-LD metadata on every search result page for a single dataset for more than five million datasets.

### Metadata via HTML Meta Tags

Data repositories *should* offer machine-readable metadata on landing pages using Highwire, PRISM^25^, and/or Dublin Core HTML meta tags. These HTML meta tags are currently the preferred method of reference managers to extract the persistent identifier or full citation metadata from landing pages, as reference managers currently don’t routinely support schema.org/JSON-LD metadata extraction.

Example

<meta name="DC.identifier" content="doi:10.1594/PANGAEA.727206" scheme="DCTERMS.URI"/>

<meta name="DC.title" content="Landings of European lobster (Homarus gammarus) and edible crab (Cancer pagurus) from 1615 to 2009, Helgoland, North Sea"/>

<meta name="DC.creator" content="Schmalenbach, Isabel"/>

<meta name="DC.creator" content="Mehrtens, Folke"/>

<meta name="DC.creator" content="Janke, Michael"/>

<meta name="DC.creator" content="Buchholz, Friedrich"/>

<meta name="DC.publisher" content="PANGAEA"/>

<meta name="DC.date" content="2011–01–28" scheme="DCTERMS.W3CDTF"/>

<meta name="DC.type" content="Dataset"/>

### Metadata via downloadable file in standard bibliographic format

Repositories *should* provide a download link in a common bibliographic format – e.g. bib (BibTeX file format) and/or. ris (RIS file format) – on the landing page of the dataset. The file should include all metadata required for a data citation.

Example: BibTeX

@data{25240_2014,

author={Figueiredo, Dalson and Rocha, Enivado and Paranhos, Ranulfo and Alexandre, José},

publisher={Harvard Dataverse},

title={How can soccer improve statistical learning?},

year={2014}, doi={10.7910/DVN/25240},

url={https://doi.org/10.7910/DVN/25240}}

Example: RIS

TY - DATAT1 - How can soccer improve statistical learning?

A1 - Figueiredo, Dalson

A1 - Rocha, Enivaldo

A1 - Paranhos, Ranulfo

A1 - Alexandre, José

Y1 - 2014

DO - 10.7910/DVN/25240

UR - https://doi.org/10.7910/DVN/25240

ER -

### Content negotiation for machine-readable metadata

Persistent identifiers expressed as HTTP URI *must* by default resolve to the landing page for that dataset (see guideline #3). Data repositories and identifier service providers such as identifiers.org, N2T or DataCite in addition *may* implement HTTP content negotiation^26^ for the persistent identifier expressed as HTTP URI, returning machine readable metadata in various formats. Content negotiation is for example supported by identifiers.org and DataCite and can return metadata in RDF-XML, BibTeX, schema.org and other metadata formats.

Example: Image Attribution Framework (IAF)

curl -H "Accept: application/xml"

http://iaf.virtualbrain.org/lp/10.18116/C6WC71

In addition, the HTML version of this page has a link to the XML (available without content negotiation at http://iaf.virtualbrain.org/lp/xml/10.18116/C6WC71).

Examples: DataCite

curl -LH "Accept: application/ld+json" http://doi.org/10.5061/DRYAD.8290N

curl -LH "Accept: application/vnd.citationstyles.csl+json"

http://doi.org/10.5061/DRYAD.8290N

Metadata in application/vnd.citationstyles.csl + json format are used as input by many reference managers, e.g. Zotero or Mendeley.

### Support HTTP link headers

The persistent identifier (see guideline #2) and available content negotiation options (see guideline #9) *may* be provided in a HTTP link header^27^. This facilitates discovery of content negotiation options and makes it easier to fetch the identifier from large landing pages, as only a HTTP head request is needed).

Example

curl -I https://search.datacite.org/works/10.5061/dryad.q447c/3

HTTP/1.1 200 OK

Content-Type: text/html;charset=utf-8

Status: 200 OK

Link:<https://doi.org/10.5061/dryad.q447c/3>; rel="identifier",

<https://doi.org/10.5061/dryad.q447c/3>; rel="describedby";

type="application/vnd.datacite.datacite+xml",

<https://doi.org/10.5061/dryad.q447c/3>; rel="describedby";

type="application/ld+json",

<https://doi.org/10.5061/dryad.q447c/3>; rel="describedby";

type="application/vnd.citationstyles.csl+json",

<https://doi.org/10.5061/dryad.q447c/3>; rel="describedby";

type="application/x-bibtex"

## Discussion

This document provides a roadmap for scholarly data repositories to implement support for data citation. Most if not all Required steps have already been implemented by many data repositories, and little if any work is needed by them to fully support the Joint Declaration of Data Citation Principles. More work is still needed to implement the Recommended steps, including support for schema.org/JSON-LD markup embedded into dataset landing pages. Data repositories that have implemented the required and recommended steps might be interested to look into the Optional steps for extra data citation support.

The Data Citation Implementation Pilot and this document focus on data citation support in scholarly data repositories. Using persistent identifiers, standard machine-readable metadata and landing pages of course not only supports data citation, but also facilitates data discovery. Data discovery requires more specific metadata than the metadata needed for data citation, and it is facilitated by a central index of all datasets. The NIH BD2K bioCADDIE project, of which the Data Citation Implementation Pilot is a small part, has developed standard metadata for biomedical data with DATS, and on a central index to search a large number of biomedical datasets with DataMed (https://datamed.org/). The European ELIXIR (https://www.elixir-europe.org/ project (https://www.elixir-europe.org/) in life sciences, and DataCite (all disciplines), are also working on standard metadata and a search index for data discovery. Both Elixir and DataCite are closely collaborating with bioCADDIE in these activities. The NIH Data Commons Pilot, which began in 2018, will further extend this work, and several of the authors of this document have participated in this project^28^.

The data citation roadmap for scholarly data repositories described in this document is an important step towards full data citation support by data repositories. Going forward, a lot of work is still needed to fully implement these guidelines, and ongoing coordination amongst data repositories, publishers and other important stakeholders will be essential in this activity.

## Methods

This roadmap was developed based on numerous discussions of the DCIP Repositories Early Adopters Expert Group, led by Martin Fenner and Mercè Crosas, including two in-person workshops in February (Boston) and June (San Diego) 2016, and in close coordination with the other DCIP expert groups. The resulting guidelines have been widely circulated since their first publication as a preprint on bioRXiv^29^. A course on the guidelines and how to implement them, was held at the FORCE11 Scholarly Communication Institute (FSCI) in August of 2017. The course instructors were Martin Fenner and Gustavo Durand, with guest speaker Natasha Noy from the schema.org initiative.

At the conclusion of the course, a hackathon was coordinated by Fenner and Durand, with Noy helping in schema.org metadata integrations. This hackathon was open to the course participants as well as other interested attendees at FSCI. Small teams that included staff from several data repositories were formed and each worked on implementing at least one of the ten guidelines for their respective data repositories. Overall, the hackathon focused on machine-readable metadata in landing pages, specifically in schema.org JSON-LD, and some repositories had implemented schema.org support by the end of the hackathon.

The course and hackathon provided valuable feedback regarding the guidelines; and served as both a propagation mechanism for the guidelines and a means of informal validation of current status with practitioners. Based on discussions at that time, with technologists from the sixteen repositories represented at our workshop, most of them had already implemented guidelines 1–6, and all had implemented guideline 1. Most had plans to implement all the guidelines, whether required, recommended, or optional. This led us to expect that many data repositories may already follow the *required* recommendations but need further work to implement the *recommended* or *optional* ones.

To follow up on the implementation of the guidelines, we looked at the adoption of guideline 8 six months after the above workshop and 12 months after the publication of the preprint. Guideline 8 recommends embedding machine-readable metadata in dataset landing pages, using the schema.org metadata standard. This particular guideline was clearly high on the priority list for implementation at the FSCI course, and its implementation was the main topic at the hackathon.

**Fig. 3:**
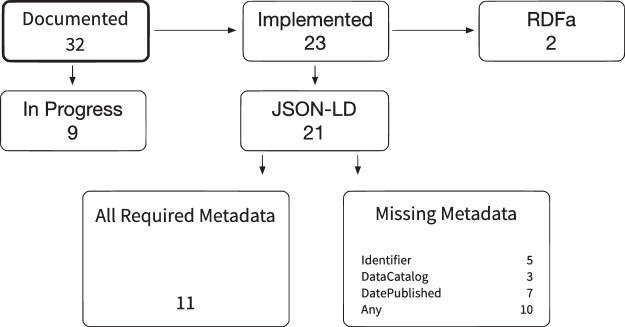
Implementation status of Schema.org metadata in repository landing pages.

We reached out to the data sharing community using mailing lists, social media and personal communications starting in January 2018, and collected information about implementations using a CSV file hosted in a GitHub repository^30^. We found 32 data repositories embedding schema.org metadata as of May 2018, and information for 8 repositories was added by these repositories via GitHub pull request. We collected information about the inclusion of the metadata fields that were required or recommended in our repository recommendations, included URLs for examples were available, and we checked whether all required metadata were included. These results are summarized in Fig. 3.

While the number of repositories in this sample is still small, we can see that a number of repositories not only are embedding schema.org metadata in their landing pages, but that half of them support all required metadata described in this document. The most frequently missing metadata elements are identifier and includedInDataCatalog/publisher and, surprisingly, publicationDate (which could also be the publication year). All these metadata elements can be easily added, but more work is probably needed to provide feedback to these early adopters. Two repositories implemented schema.org using RDFa. While this is an accepted serialization format for schema.org metadata, this document recommends standardization on JSON-LD to simplify tool development, e.g. reference manager support. We are also seeing a broad range of recommended metadata implemented, and that will help with data discovery, e.g. via Google Dataset Search. Recent software releases will also be helpful, including DataCite’s new link checker^31^. We believe the development and release of such tools by major providers will further incentivize repositories to follow the guidelines in this article.

In addition to the implementations in repository landing pages noted earlier, we are also seeing implementations in supporting services for data repositories: the Dataverse repository platform added schema.org support in December 2017^32^, and DataCite added support for direct DOI registration using schema.org metadata embedded in the dataset landing page in May 2018^33^.

## Data Availability

We compiled a dataset through community consultation which lists data repositories that embed schema.org metadata. The dataset is available as a CSV file within the Zenodo repository^30^.
